# A Focus on Two Electrokinetics Issues

**DOI:** 10.3390/mi11121028

**Published:** 2020-11-24

**Authors:** Cheng Dai, Ping Sheng

**Affiliations:** Department of Physics, HKUST, Clear Water Bay, Kowloon, Hong Kong, China; cdaiaa@connect.ust.hk

**Keywords:** Poisson-Boltzmann equation, electrophoresis, holistic approach, electrophoretic drag coefficient, inner flow field vortices

## Abstract

This review article intends to communicate the new understanding and viewpoints on two fundamental electrokinetics topics that have only become available recently. The first is on the holistic approach to the Poisson–Boltzmann equation that can account for the effects arising from the interaction between the mobile ions in the Debye layer and the surface charge. The second is on the physical picture of the inner electro-hydrodynamic flow field of an electrophoretic particle and its drag coefficient. For the first issue, the traditional Poisson–Boltzmann equation focuses only on the mobile ions in the Debye layer; effects such as charge regulation and the isoelectronic point arising from the interaction between the mobile ions in the Debye layer and the surface charge are left to supplemental measures. However, a holistic treatment is entirely possible in which the whole electrical double layer—the Debye layer and the surface charge—is treated consistently from the beginning. While the derived form of the Poisson–Boltzmann equation remains unchanged, the zeta potential boundary condition becomes a calculated quantity that can reflect the various effects due to the interaction between the surface charges and the mobile ions in the liquid. The second issue, regarding the drag coefficient of a spherical electrophoretic particle, has existed ever since the breakthrough by Smoluchowski a century ago that linked the zeta potential of the particle to its mobility. Due to the highly nonlinear mathematics involved in the electro-hydrodynamics inside the Debye layer, there has been a lack of an exact solution for the electrophoretic flow field. Recent numerical simulation results show that the flow field comprises an inner region and an outer region, separated by a rather sharp interface. As the inner flow field is carried along by the particle, the measured drag is that at the inner/outer interface rather than at the solid/liquid interface. This identification and its associated physical picture of the inner flow field resolves a long-standing puzzle regarding the electrophoretic drag coefficient.

## 1. Introduction

Electrokinetics is a classical field of study with a long history that goes back at least two centuries. However, it is still a vibrant area of investigation mainly because of the potential applications of the various associated phenomena, as well as the richness of the field and the newly available tools. This tutorial review focuses on two issues that occupy a rather fundamental position in this classical field—a holistic approach to the Poisson–Boltzmann (PB) equation that can account for the interaction between the mobile ions and the surface charge layer, and the electro-hydrodynamics of the Debye layer and the hydrodynamic drag of a spherical electrophoretic particle. In Section 2, we present the theoretical equations involved in the study of these two issues—the Poisson–Nernst–Planck (PNP) equations coupled with the Navier-Stokes (NS) equation—followed by a re-derivation of the PB equation from the holistic approach in Section 3 with an explicit methodology to calculate the boundary condition that replaces the traditional zeta potential. Two predictions of the holistic approach, the isoelectronic point of the surface charge and the surface reactivity value for the charge separation process, are shown to be in good agreement with the experiments. In Section 4, we describe the essential results of an extensive theoretical and experimental study on the electrophoretic drag that led to a new, more complete physical picture of electrophoresis and its flow field. We conclude in Section 5 with a short concluding remark. In order not to digress from the main line of exposition, we leave out the technical details on the experiments and simulations that are available in the relevant references.

## 2. The Poisson–Nernst–Planck (PNP) Equations

Each ion in a fluid experiences electrical forces due to other ionic charges and the externally applied electric field, as well as the random collisions by water molecules. The fluid ions are also carried along by the fluid’s velocity field when there is a flow. The resulting ion dynamics and their constraints, such as the conservation of electrical charges, can be mathematically described below by the PNP equations [1,2,3,4] coupled with the Navier–Stokes equation:

(1a) $$\frac{\partial n_{\pm }}{\partial t}=-\nabla •J_{\pm },$$

(1b) $$J_{\pm }=-D_{ion}\left(\nabla n_{\pm }(r)\pm \frac{e}{k_{B}T}n_{\pm }(r)\nabla \varphi (r)\right)+n_{\pm }(r)u(r),$$

(1c) $$\nabla^{2}\varphi =-\frac{e(n_{+}-n_{-})}{\varepsilon_{f}\varepsilon_{o}},$$

(1d) $$\rho_{l}\frac{\partial u}{\partial t}=\eta \nabla^{2}u-e(n_{+}-n_{-})\nabla \varphi -\nabla P,$$

(1e) ∇•u=0.

Here φ stands for the electrostatic potential, which can include both the potential arising from the applied electric field as well as that from the other ionic charges; $n_{\pm }$ denotes the positive and negative ion densities; *e* denotes the absolute value of the electronic charge; $k_{B}$ is the Boltzmann constant; and *T* denotes temperature. Equation (1a) expresses the conservation of charges in which $J_{\pm }$ denotes the ionic current density. In the current density expression, Equation (1b), there are diffusive, electrical convective and flow convective components, where the Einstein relation has been used to express the electrical conductivity in terms of the diffusion constant, and ***u*** is the fluid velocity. The valence of all ions is taken to be one. Here, we use the value of $D_{ion}=9.32\times 10^{-9}m^{2}/s$ [5]. It should be noted that the effect of random collisions on the ions is expressed by the diffusive dynamics in the ionic current density. Electrical interactions are completely described by the Poisson equation, Equation (1c), where the dielectric constant of water, $\varepsilon_{f}\varepsilon_{0}$, is taken to be $\varepsilon_{f}=80$, with $\varepsilon_{0}=8.85\times 10^{-12}F/m$. Equation (1d) is the Navier–Stokes (NS) equation, where we have neglected the nonlinear inertial term $\rho_{l}(u•\nabla )u$; $\eta =1\times 10^{-3}$ Pa.s is the shear viscosity, taken to be that of water; $\rho_{l}$ is the density of water and *P* denotes pressure. Equation (1e) expresses the incompressibility condition of the fluid. The PNP equations, when coupled with the NS equation, constitute the mathematical basis of electrokinetics. However, only the PNP equations are relevant to the holistic approach to the Poisson-Boltzmann equation. The whole set is required for the simulation of the electrophoresis phenomenon.

A careful examination of Equations (1a–e) shows that all the equations are coupled to each other. The kinematic boundary conditions for the PNP and NS equations are as follows. At the fluid/solid interface, we have u=0 and $J_{\pm }•\hat{n}$ = 0, where $\hat{n}$ is the unit vector normal to the interface. These conditions guarantee the conservation of both *n_+_* and *n*_−_ and thus the overall charge neutrality of the system. The electrical boundary conditions at the solid/liquid interface are the most important, as they give rise to the electrical double layer (EDL) and thus the electrokinetic phenomena. Traditionally, these can be either the Dirichlet type boundary conditions, in which a constant zeta potential is specified, or the Neumann type (Gouy–Chapman) boundary conditions.

### Poisson–Boltzmann Equation as the Static Limit of the PNP Equations

By setting $J_{\pm },u=0$, we have from Equation (1b)

(2a) $$\nabla n_{\pm }\pm \frac{e}{k_{B}T}n_{\pm }\nabla \varphi =0.$$

Equation (2a) can be integrated to yield

(2b) $$n_{\pm }=\alpha_{\pm }\exp[\mp e\varphi /k_{B}T].$$

As we can see from Equation (2b), the Boltzmann distribution of the ionic densities arises from a detailed balance between the drift current and the diffusive countercurrent. Here, $\alpha_{\pm }$ represents the integration constants. By setting $n_{\pm }=n^{\infty }$, the volume–average ion density in the bulk limit, and substituting this solution into the Poisson equation, we obtain the local net charge density $\rho_{E}$ as

(3a) $$\rho_{E}=-e(n_{+}-n_{-})=-2en^{\infty }\sinh\left(\frac{e\varphi }{k_{B}T}\right)\;,$$

so that

(3b) $$\nabla^{2}\bar{\varphi }=\frac{1}{\lambda_{D}^{2}}\sinh\left(\bar{\varphi }\right),$$

where $\bar{\varphi }=e\varphi /k_{B}T$, with $\lambda_{D}=\sqrt{\varepsilon k_{B}T/(2e^{2}n^{\infty })}$ being the Debye length. Equation (3b) is the Poisson–Boltzmann equation. Since $\sinh(\bar{\varphi })$ is monotonic, the volume integral of the net charge density on the right-hand side of Equation (3b) must have a finite value; i.e., the Debye layer is not charge-neutral. In fact, the Debye layer represents only half of the EDL, with the other half being the surface charge on the liquid/solid interface. The system must be electrically neutral overall, as a net charge can only exist in special cases—mostly during a short transient time period.

In what follows, it will be seen that by starting from the PNP equations and requiring the system to be electrically neutral, one can arrive at a charge-conserved PB (CCPB) equation [5,6,7]. By further adding an energy-based charge separation mechanism with a small spatial footprint within the computational domain of the PNP equations, we can recover the form of Equation (3b) via a chemical potential transformation. The recovered PB equation has an associated boundary condition that requires explicit evaluation, and which contains all the effects arising from the electrical interaction between the Debye layer and the surface charge layer under the influence of diffusive dynamics.

## 3. Holistic Approach to the Poisson–Boltzmann Equation

### 3.1. Charge-Conserved Poisson–Boltzmann Equation

We first define a computational domain that has cylindrical symmetry, with the two ends of the cylinder being at infinity. The radius of the cylinder is denoted by *a*. As the ionic fluid is overall charge-neutral, from Equation (2b) we must have

(4) $$\alpha_{-}\int_{V}dx\exp[+e\varphi /k_{B}T]=n^{o}V=\alpha_{+}\int_{V}dx\exp[-e\varphi /k_{B}T],$$

where we have introduced the value $n^{o}$, which differs from $n^{\infty }$ by the fact that $n^{o}$ also includes the surface charge density in the following manner:

(5) $$n_{\pm }^{o}=n_{\pm }^{\infty }+2\frac{\sigma_{\pm }}{a}.$$

Equation (5) is obtained by requiring $n_{\pm }^{o}(\pi a^{2}L)=n_{\pm }^{\infty }(\pi a^{2}L)+\sigma_{\pm }(2\pi aL)$, where *L* denotes the length of the liquid channel. The fact that $n_{\pm }^{O}>n_{\pm }^{\infty }$ is due to the interfacial dissociation mechanism that increases the overall ionic density from the bulk ion density. The latter is noted to be governed by the equilibrium law of mass action; e.g., for *H*^+^ and *OH*^−^ ions in water.

From Equations (2b) and (4), we have

(6a) $$n_{\pm }=n^{o}\frac{\exp[\mp e\varphi /k_{B}T]}{\frac{1}{V}\int_{V}dx\exp[\mp e\varphi /k_{B}T]},$$

which leads to the following CCPB [5,6,7] in cylindrical coordinates:

(6b) $$\frac{1}{r}\frac{\partial }{\partial r}\left(r\frac{\partial \varphi }{\partial r}\right)=\frac{ea^{2}n^{o}}{2\varepsilon }\left[\frac{\exp(e\varphi /k_{B}T)}{\int_{0}^{a}r\exp(e\varphi /k_{B}T)dr}-\frac{\exp(-e\varphi /k_{B}T)}{\int_{0}^{a}r\exp(-e\varphi /k_{B}T)dr}\right].$$

As we can see, the integration of the right-hand side of Equation (6b) would yield zero, ensuring the overall charge neutrality of the system. However, this is an integral–differential equation that is difficult to solve. Below, we show that there is a chemical potential transform [5,7] which can convert Equation (6b) into two equations, in which one has a form that can be converted into the PB equation while the other is the definition of a chemical potential that is based on the constraint of global charge neutrality. When solved together, they not only guarantee overall charge neutrality, but also yield all the consequences of electrical interactions between the mobile ions in the Debye layer and the surface charge layer.

### 3.2. The Chemical Potential Transform

We would like to separate Equation (6b) into two equations that, when considered together, would ensure the overall charge neutrality of the system, while each on its own is easier to handle than Equation (6b). The first step is to re-write Equation (6b) in the following form:

(7a) $$\frac{1}{r}\frac{\partial }{\partial r}\left(r\frac{\partial \varphi }{\partial r}\right)=\frac{ea^{2}n^{o}}{2\varepsilon }\left\{\exp[e(\varphi -\mu )/k_{B}T]-\exp[-e(\varphi -\mu )/k_{B}T]\right\},$$

where the chemical potential μ is defined by the condition of overall charge neutrality; i.e., the integral of the right-hand side of Equation (7a) should be zero:

(7b) $$\exp(-e\mu /k_{B}T)\int_{0}^{a}r\exp(e\varphi /k_{B}T)dr-\exp(e\mu /k_{B}T)\int_{0}^{a}r\exp(-e\varphi /k_{B}T)dr=0.$$

That means that the chemical potential is given by the expression

(7c) $$\mu =\frac{k_{B}T}{2e}\ln\left\{\frac{\int_{0}^{a}r\exp(e\varphi /k_{B}T)dr}{\int_{0}^{a}r\exp(-e\varphi /k_{B}T)dr}\right\}.$$

It can be seen that Equation (7a) is now at the same level of mathematical difficulty as the PB equation, but constrained by the condition of overall charge neutrality, as shown in Equation (7c).

### 3.3. Surface Potential Trap

To model an EDL, it is necessary to introduce a charge separation mechanism in the computational domain [5,7]. In this context, we observe that a natural phenomenon occurs because invariably it lowers the (free) energy of the system. Thus, in order to model the charge separation process in our cylindrical computational domain, we wish to introduce a surface potential trap at the circumference of the cylinder that has the following features:

(1) It should be charge-neutral, so that the trap does not add or subtract charges from the system.
(2) It should attract charges of a definite sign and repel those with the opposite sign.
(3) It should have a small spatial footprint so as to model the thin surface charge layer. That is, the force exerted by the potential trap on the attracted (surface) charges exists only within a small region of space close to the solid boundary. The bulk ions only feel the electrostatic attraction/repulsion of the surface charges inside the trap, but not those from the trap itself.

Since the potential trap is within the computational domain, it is subjected to the same diffusive dynamics and charge interactions as dictated by the PNP equations. Therefore, the magnitude of the surface charge density is a function of the depth of the potential trap, as well as the concentration of the relevant ions in the bulk region. In regard to the latter, it is a fact of nature—e.g., for the silica/water interface—that the solid surface is only reactive to the *H*^+^ and *OH*^−^ ions, but not to other ions that might result from dissociated ions from added salts; e.g., *Na*^+^ and *Cl*^−^. Below, we first give the functional form of the surface potential trap, followed by a solution approach to the Poisson equation that can exclude all the other ions except the *H*^+^ and *OH*^-^ ions in the potential trap region [7].

#### 3.3.1. Form of the Surface Potential Trap

The trap function f(r), where *r* is the radial coordinate, has two parameters—the height of the trap γ and its width Δ:

(8a) $$f(r)=\frac{\gamma }{2}\left(1+\cos\frac{\pi (r-a)}{\Delta }\right),\;a-\Delta \leq r\leq a$$

(8b) f(r)=0,      0≤r≤a−Δ.

The width of the surface potential trap, Δ, is set to be the length of a hydrogen bond; i.e., about 8 Å. To verify that the trap function does not add or subtract any net charges to the system, we simply evaluate $\nabla^{2}f(r)=-\rho_{trap}/\varepsilon$. One can easily verify that the integration of $\rho_{trap}$ over the domain a−Δ≤r≤a is zero. Here, $\rho_{trap}$ is regarded as external to the system. The height of the potential trap is generally taken to be on the order of +0.5 eV; i.e., on the same order as that for a hydrogen bond. The width of the potential trap is smaller than 1 nm, but a finite width is necessary in order for the diffusive dynamics to be effective in regulating the surface charge.

#### 3.3.2. Making the Potential Trap Selective in Terms of the Types of Ions

**Figure 1:**
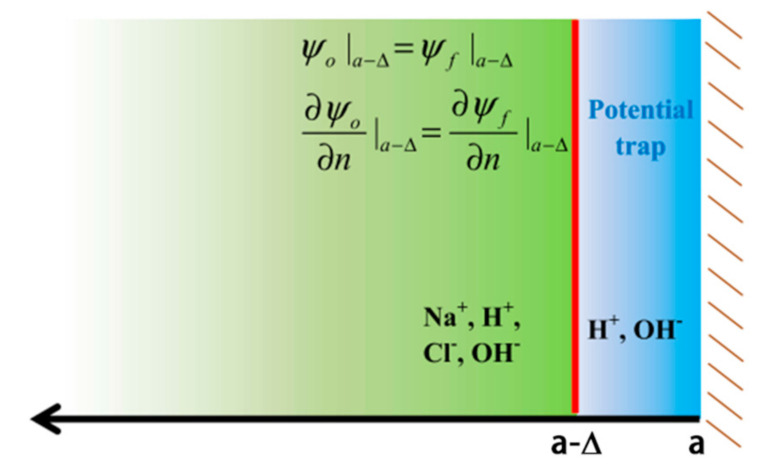
A schematic illustration of the sub-domains in the solution of the charge-conserved Poisson–Boltzmann (CCPB) equation, colored in green and blue. The solutions in the two regions are linked together by the two electrostatic boundary conditions at the interface, denoted by the red line. This division of the computational domain is to ensure that no surface-non-specific salt or buffer ions can enter the surface potential trap. The latter has a width of ∆. This physical condition is especially important in modeling the isoelectronic point and its related properties. Adapted from [7].

The introduction of a surface potential trap *f* means that the potential function φ should be written as φ(r)=ψ(r)+f(r), where φ(r)=ψ(r) in the domain 0≤r≤a−Δ. In order to make the potential trap selective, we further divide the solution of ψ(r) into two spatial regions, as shown in Figure 1. We denote the potential in the spatial region 0≤r≤a−Δ as $\psi_{o}$ and that in the region of the potential trap as $\psi_{f}$. Thus, the total potential in the trap region is $\psi_{f}+f$. As indicated in Figure 1, the interfacial boundary conditions connecting the potentials of the two spatial regions are the continuity of the ψ function and its normal derivative at r=a−Δ. It should be noted that, in Equations (7a–c), both the value of *f* and its normal derivative vanish at r=a−Δ; thus, it does not enter the connection boundary condition. This approach [7] assures the selectivity of the potential trap to only the surface-specific *H*^+^ and *OH*^−^ ions when implemented with the mathematical form of the CCPB given below.

#### 3.3.3. The Modified form of the CCPB and the Chemical Potential Transform

The introduction of the surface potential trap, in conjunction with the two-region approach to the solution of the Poisson equation, means that the CCPB must also be divided into two spatial regions as follows. In the following, we will assume that the salt *NaCl* is added. In anticipation of the chemical potential transform, we write the equations in the two regions as follows.

In the region 0≤r≤a−Δ, we have

(9a) $$\frac{1}{r}\frac{\partial }{\partial r}\left(r\frac{\partial \psi_{o}}{\partial r}\right)=\frac{e}{\varepsilon }\left\{\begin{array}{l} n_{Cl^{-}}^{\infty }\exp[e(\psi_{o}-\mu )/k_{B}T]+n_{OH^{-}}^{\infty }\exp[e(\psi_{o}-\mu )/k_{B}T]\\ -n_{Na^{+}}^{\infty }\exp[-e(\psi_{o}-\mu )/k_{B}T]-n_{H^{+}}^{\infty }\exp[-e(\psi_{o}-\mu )/k_{B}T] \end{array}\right\}.$$

In the region a−Δ≤r≤a, we have

(9b) $$\frac{1}{r}\frac{\partial }{\partial r}\left(r\frac{\partial \psi_{f}}{\partial r}\right)=\frac{e}{\varepsilon }\left\{\begin{array}{l} n_{OH^{-}}^{\infty }\exp[e(\psi_{f}-\mu +f)/k_{B}T]\\ -n_{H^{+}}^{\infty }\exp[-e(\psi_{f}-\mu +f)/k_{B}T] \end{array}\right\}.\;$$

The boundary conditions are given by

(9c) $$\begin{array}{l} \psi_{f}{|}_{r=a}=0\\ {\left[\begin{array}{l} \psi_{o}=\psi_{f}\\ \frac{\partial \psi_{o}}{\partial r}=\frac{\partial \psi_{f}}{\partial r} \end{array}\right]}_{r=a-\Delta } \end{array}.$$

By requiring the integrated ionic charge densities on the right-hand sides of Equations (9a,b) to be zero so as to ensure overall charge neutrality—a mathematical step similar to that shown by Equation (7b) but now involving the summation of the charges in two regions—we obtain the expression for the chemical potential as

(9d) $$\mu =\frac{k_{B}T}{2e}\ln\left\{\frac{\int_{0}^{a-\Delta }\{n_{OH^{-}}^{\infty }\exp(e\psi_{o}/k_{B}T)+n_{Cl^{-}}^{\infty }\exp(e\psi_{o}/k_{B}T)\}rdr+\int_{a-\Delta }^{a}n_{OH^{-}}^{\infty }\exp[e(\psi_{f}+f)/k_{B}T]rdr}{\int_{0}^{a-\Delta }\{n_{H^{+}}^{\infty }\exp(-e\psi_{o}/k_{B}T)+n_{Na^{+}}^{\infty }\exp(-e\psi_{o}/k_{B}T)\}rdr+\int_{a-\Delta }^{a}n_{H^{+}}^{\infty }\exp[-e(\psi_{f}+f)/k_{B}T]rdr}\right\}.$$

A beneficial property of Equations (9a–d) is that an additive constant potential would mean only a constant shift in both ψ and μ, leaving ψ−μ invariant and thus having no physical implications.

It should be noted that, in the above, all the ionic charge densities are given in terms of the experimentally measurable $n_{}^{\infty }$ for each ionic species in order to facilitate comparisons of the theory predictions with experimental results. Here, we have the condition $n_{}^{\infty }=n_{-}^{\infty }=n_{OH^{-}}^{\infty }+n_{Cl^{-}}^{\infty }=n_{Na^{+}}^{\infty }+n_{H^{+}}^{\infty }=n_{+}^{\infty }$. The values of $n_{H^{+}}^{\infty }$ and $n_{OH^{-}}^{\infty }$ are noted to be constrained by the law of mass action, $n_{H^{+}}^{\infty }(M)•n_{OH^{-}}^{\infty }(M)=10^{-14}(M^{2})$, where *M* denotes molar concentration. The law of mass action governs the equilibrium reaction rate, and in this case, it applies to the $H^{+}$ and $OH^{-}$ ions in acid or alkaline solutions. As mentioned previously, the existence of the charge separation process that is realized by the surface potential trap potential would increase the overall charge density from $n^{\infty }$, the resulting $n_{\pm }^{o}$ be evaluated as

(10) $$\begin{array}{ll} n^{o} & =n_{-}^{o}=\frac{2}{a^{2}}\int_{0}^{a}\{n_{OH^{-}}^{\infty }\exp[e(\psi -\mu +f)/k_{B}T]+n_{Cl^{-}}^{\infty }\exp[e(\psi -\mu )/k_{B}T]\}rdr\\ & =n_{+}^{o}=\frac{2}{a^{2}}\int_{0}^{a}\{n_{H^{+}}^{\infty }\exp[-e(\psi -\mu +f)/k_{B}T]+n_{Na^{+}}^{\infty }\exp[-e(\psi -\mu )/k_{B}T]\}rdr \end{array}.$$

It is noted that if there is no surface potential trap, then *f* = 0, $\psi_{f}=\psi_{o}=\psi$ and ψ−μ=0, which would lead directly to $n_{\pm }^{o}=n_{\pm }^{\infty }=n_{OH^{-}}^{\infty }+n_{Cl^{-}}^{\infty }=n_{Na^{+}}^{\infty }+n_{H^{+}}^{\infty }$, as it should.

### 3.4. Re-Derivation of the Poisson–Boltzmann Equation

From Equation (9a), only one step is required to obtain the form of the PB equation. Since the PB equation applies only to the liquid region, i.e., in the region 0≤r≤a−Δ, hence by a slight re-arrangement of Equation (9a), we obtain

(11) $$\begin{array}{l} \frac{1}{r}\frac{\partial }{\partial r}\left(r\frac{\partial \psi_{o}}{\partial r}\right)=\frac{e}{\varepsilon }\left\{n_{-}^{\infty }\exp\left[\frac{e(\psi_{o}-\mu )}{k_{B}T}\right]-n_{+}^{\infty }\exp\left[-\frac{e(\psi_{o}-\mu )}{k_{B}T}\right]\right\}\\ =\frac{2en_{}^{\infty }}{\varepsilon }\sin h\left[\frac{e(\psi_{o}-\mu )}{k_{B}T}\right] \end{array},$$

where we have used the fact that $n^{\infty }=n_{-}^{\infty }=n_{+}^{\infty }$. By multiplying both sides by $e/k_{B}T$ to non-dimensionalize the potential, as well as by defining

(12a) $${\bar{\varphi }}^{\left(PB\right)}=\frac{e}{k_{B}T}(\psi_{o}-\mu ),$$

we obtain the PB equation in the cylindrical coordinate:

(12b) $$\frac{1}{r}\frac{\partial }{\partial r}\left(r\frac{\partial {\bar{\varphi }}^{\left(PB\right)}}{\partial r}\right)=\frac{1}{\lambda_{D}^{2}}\sin h{\bar{\varphi }}^{\left(PB\right)}.$$

Since the surface potential trap is only 8 Angstroms in width, the potential difference between r=a and r=a−Δ is negligible (a fraction of a millivolt in practice); thus, we can take the value of ψ at r=a−Δ to be essentially that at r=a, which is specified to be 0 as in Equation (9c). That means that the boundary condition at r=a−Δ for the PB equation is given by

(12c) $${\bar{\varphi }}^{\left(PB\right)}{|}_{r=a-\Delta }=-\frac{e\mu }{k_{B}T}=-\bar{\mu }$$

at the liquid/solid interface. Thus, *the negative of the chemical potential replaces the zeta potential as the boundary condition for the PB equation*. Since the chemical potential is a part of the solution (instead of being a given input as in the case of the traditional zeta potential) to be evaluated in accordance with Equation (9d), it contains all the information pertaining to the electrical interaction between the surface charge layer and the Debye counterions in the liquid.

### 3.5. Evaluation of the Surface Charge Density and the Zeta Potential

The surface charge density *S* can be obtained by evaluating the net charges inside the potential trap:

(13) $$S=\frac{1}{a}\left[\left(n_{H^{+}}^{\infty }\right)\int_{a-\Delta }^{a}\exp[-e(\psi_{f}-\mu +f)/k_{B}T]rdr-\left(n_{OH^{-}}^{\infty }\right)\int_{a-\Delta }^{a}\exp[e(\psi_{f}-\mu +f)/k_{B}T]rdr\right].$$

The value of *S* should always be larger than $\sigma_{-}$ since the latter represents the average value between the Debye layer region and the potential trap region, but the Debye layer region is characterized by a deficit of *OH*^-^ ions. As for the zeta potential ζ, we would like to define it as the potential increase between the center of the channel and the liquid/solid interface:

(14) $$\zeta =-\frac{2}{a^{2}}\int_{0}^{a-\Delta }\psi_{o}(r)rdr.$$

**Figure 2:**
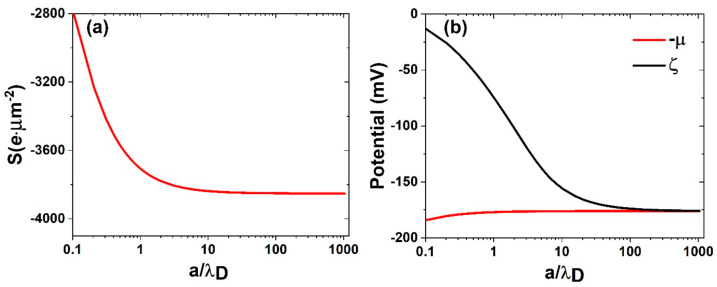
(**a**) The magnitude of the surface charge plotted as a function of the channel radius, in units of the Debye length. (**b**) The zeta potential and the negative of the chemical potential plotted as a function of the normalized channel radius. The calculated case is for pH 7, with no salt added. The energy height of the potential trap used is γ = 510 mV. It is seen that, in the large channel limit, the two quantities converge to a common value. Adapted from [7].

In Figure 2a, we plot the surface charge as a function of the channel radius, normalized by the Debye length. In Figure 2b, the zeta potential and the negative of the chemical potential are similarly plotted as a function of the normalized channel radius. It is seen that in the large channel radius limit the red and black curves converge, but as the channel radius decreases the zeta potential decreases in magnitude. This is easy to understand, as the Debye length defines the decay length of the potential value from the solid/liquid interface. As the channel radius decreases, the potential decay can no longer reach zero as in a large channel, and the zeta potential as defined by Equation (14) decreases as a result, in conjunction with the net positive ions in the Debye layer. This has to be accompanied by a decrease in the surface charge density since the EDL has to maintain overall charge neutrality. Thus, when the channel diameter decreases below the Debye layer thickness, the surface charge separation process is suppressed because the diffusive dynamics dictates that only a small difference in ionic concentration can exist over a small distance, with the attendant effect of decreased electrokinetic activity.

### 3.6. Predictions of the Holistic Approach

Here, we would like to describe two predictions of the holistic approach. The first is the phenomenon of the isoelectronic point of the surface charge density, which occurs when the liquid is highly acidic; i.e., with a high concentration of *H*^+^ ions. The second is the surface reactivity—the so-called pK value.

#### 3.6.1. Prediction 1: Isoelectronic Point for the Surface Charge Density

**Figure 3:**
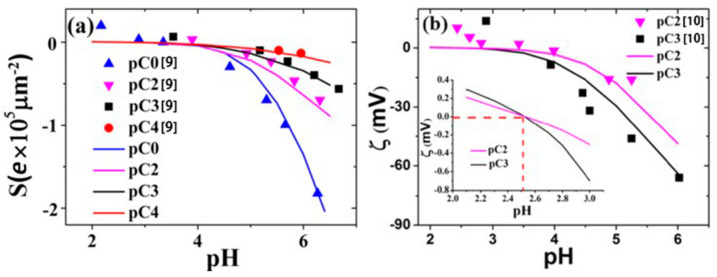
(**a**) Calculated surface charge density plotted as a function of pH values with a channel radius of 20 μm (solid lines). Experimental data [9] are shown as filled symbols. Excellent theory–experiment agreement is seen. The pC values indicate the salt concentration; i.e., those surface-non-specific ions that are excluded by the surface potential trap. (**b**) Zeta potential plotted as a function of pH values under different salt concentrations pC, with a channel radius of 20 μm. Theory predictions are shown as the solid lines, and experimental data are shown as filled symbols [10]. Semi-quantitative agreement is seen. The inset shows an enlarged view of the zeta potential at around pH 2–3. The zeta potential is seen to cross zero at the same isoelectronic point, pH 2.5, for two different salt concentrations. All the solid curves were calculated with *γ* = 510 mV. Adapted from [7].

The surface potential trap is biased against one type of the ionic charges—in the present case, the *H*^+^ ions—due to the energy consideration. However, when there is a very high concentration of *H*^+^ ions in water—i.e., in the highly acidic regime—the *H*^+^ ion concentration gradient between the bulk solution and the potential trap can be sufficiently high for the *H*^+^ ions to diffuse into the potential trap in spite of the energy barrier. In other words, here, the entropy consideration that is realized through the diffusive dynamics becomes non-negligible. When that happens, the net surface charge decreases and eventually becomes neutral. This is referred to as the isoelectronic point [8], and this phenomenon can be fully predicted by the holistic approach because all the diffusive and electrostatic interactions between the surface potential trap and the bulk are taken into account, as they both belong to the same computational domain. In Figure 3a, we show the surface charge *S* plotted as a function of the pH value of the bulk liquid solution; it is seen that *S* crosses zero at a pH of 2.5, denoting the isoelectronic point. In the figure, there are curves associated with different salt concentrations, as denoted by the pC values. Solid lines are the theory predictions from the holistic approach, and the solid symbols are the experimental data obtained through techniques such as surface titration [9]. Excellent agreement is seen. In Figure 3b, we show the theory and experimental comparisons for the zeta potential value. The theory values, shown as the solid lines, are evaluated from Equation (14). Again, good agreement is seen.

#### 3.6.2. Prediction 2: Surface Reactivity

**Figure 4:**
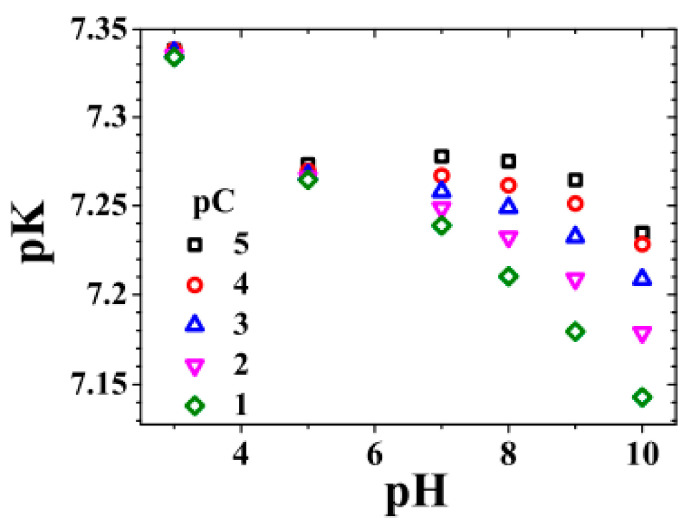
The pK values obtained from the definition of the equilibrium constant of the reaction *SiO^−^ + H^+^⇔ SiOH*. The energy height of the potential trap used is γ = 510 mV. Adapted from [7].

Another important parameter for the surface charge separation process is the so-called surface reactivity, which measures the equilibrium *H*^+^ ion concentration resulting from the surface dissociation of the silanol group on the interface between water and silica:$SiOH\Leftrightarrow SiO^{-}+H^{+}$. That parameter is defined as the pK value by the following:

(15) $$K=\frac{N_{SiO^{-}}{\left[H^{+}\right]}_{o}}{N_{SiOH}}=10^{-pK}[\mathrm{mol}/L],$$

where [*H*^+^]_o_, in units of [mol/L], is the proton local density at the outer boundary of the surface potential trap; $N_{SiO^{-}}$ and $N_{SiOH}$, both in the same unit of [nm^−2^], are the surface densities of the respective $SiO^{-}$ and SiOH groups. For $N_{SiO^{-}}$, we simply use the negative ion density (that of $OH^{-}$) inside the potential trap, integrated over its width Δ to yield the surface density; here, we are reminded of the reaction $SiOH+OH^{-}\Leftrightarrow SiO^{-}+H_{2}O$, meaning that the surface density of $OH^{-}$ should be the same as that of $SiO^{-}$. The value of [*H*^+^]_o_ can be obtained at the position just outside the surface potential trap. For the $N_{SiOH}$, one can use the total site density, 1/*v_o_*, where *v_o_* denotes the average volume occupied by a single silicon dioxide molecule, and approximate $N_{SiOH}\approx 1/v_{o}^{2/3}$ = ${\left(0.35^{3}\right)}^{-2/3}{\mathrm{nm}}^{-2}$ = 8.2 nm^−2^. This value is noted to be very close to a commonly cited literature value for nonporous, fully hydrated silica: $N_{SiOH}$ = 8 nm^−2^ [11]. The pK value so obtained is in the range of 7.14–7.28 for the pH range of 3 to 10, as shown in Figure 4. The pK value, usually considered to be independent of salt concentration and pH values (5–9), turns out to display some variation when the pH value or salt concentration increases. This agrees reasonably well with the literature-reported pK values, which can range from 6 to 8 [12,13] within the same pH range, even though there is also a report of a value of 4 [14].

### 3.7. Holistic Approach as a Platform for Further Investigations of Charged Interfacial Physics

While electrical interactions and diffusive dynamics are clearly the dominant elements of electrokinetics, other effects, such as those arising from steric repulsion between the ions in the extremely high ion concentration regime, or from chemical (quantum mechanical) factors, cannot be ruled out. To account for such other effects, one needs to have an accurate account of the dominant influence of the electrical interaction and diffusive dynamics, and here the holistic approach may serve as a platform for such further investigations. It should be noted that the holistic approach can be generalized to non-planar interfaces that may be difficult to handle otherwise.

## 4. Electrophoresis and the Electrophoretic Drag Coefficient

For a small colloidal particle whose interface with the liquid displays a charge separation phenomenon—e.g., the silica/water interface—the application of an external electric field can induce the small colloidal particle to move at a constant velocity opposite to the applied electric field direction in the case of a silica sphere. This electric-field-induced motion is referred to as electrophoresis [15,16,17], which is part of the electrokinetic family of phenomena.

When a solid particle moves steadily in liquid, it will encounter a hydrodynamic drag force F that is linearly proportional to its speed, F=γv. Here, γ is denotes the drag coefficient. When the particle is moved by a body force—e.g., the gravitational pull due to the solid/liquid density difference—the drag coefficient can be analytically derived for a solid sphere with radius *a*, denoted as the Stokes drag coefficient, $\gamma_{S}=6\pi \eta a$. However, what should the drag coefficient be for an electrophoretic particle? This question has become a controversial issue, both theoretically and experimentally. Below, we first give a heuristic description of the electrophoresis and the classical Smoluchowski argument [18], which will lead to the issue of the electrophoretic drag coefficient $\gamma_{E}$.

### 4.1. A Heuristic Understanding of the Electrophoresis Phenomenon

For an EDL induced by the charge separation at the solid/liquid interface, the surface charge layer and the Debye layer constitute an overall electrically neutral system. Thus, when an external electric field is applied to such an EDL, there is no net force. In accordance with Newton’s law of motion, in the absence of external force, the momentum of the center of mass is conserved. If the particle is initially at rest, then, with the application of an external electric field, the mobile positive ions in the Debye layer—e.g., for the silica particle—will be pulled by the applied field, thereby dragging the fluid along. Since the center of mass momentum is conserved, the solid silica particle must move in a direction opposite to the electric field. This is an account of the electrophoresis phenomenon in a nutshell.

### 4.2. The Smoluchowski Argument

The Polish physicist Marian Smoluchowski observed a century ago that if there is net charge density in a liquid, as in the case of the Debye layer, then the net charge density term would appear in both the Poisson equation and the Navier–Stokes equation [18]. In cylindrical coordinates, that means

(16) $$\frac{\partial^{2}\varphi }{\partial r^{2}}+\frac{1}{r}\frac{\partial \varphi }{\partial r}=-\frac{\rho }{\varepsilon },$$

where ρ denotes the net charge density, and

(17) $$\eta \left(\frac{\partial^{2}u_{z}}{\partial r^{2}}+\frac{1}{r}\frac{\partial u_{z}}{\partial r}\right)=\frac{\partial P}{\partial z}-\rho E_{z},$$

where $u_{z}$ is the liquid velocity along the axial direction (the *z* axis) of the cylinder and $E_{z}$ denotes the applied field along the same direction. By eliminating the charge density, the two equations can be combined:

(18) $$\left[\frac{\partial^{2}u_{z}}{\partial r^{2}}+\frac{1}{r}\frac{\partial u_{z}}{\partial r}\right]=\frac{1}{\eta }\frac{\partial P}{\partial z}+\frac{\varepsilon E_{z}}{\eta }\left[\frac{\partial^{2}\varphi }{\partial r^{2}}+\frac{1}{r}\frac{\partial \varphi }{\partial r}\right].$$

The similarity of the terms in the square brackets on the two sides of Equation (18) means that $u_{z}$ and φ must be linearly related to each other; i.e., $u_{z}(r)=C_{1}+C_{2}\varphi (r)$, where $C_{1}$ and $C_{2}$ are determined by the boundary condition stating that φ=ζ on the solid boundary plus the requirement of satisfying Equation (18). This immediately leads to the following expression:

(19) $$u_{z}=-\frac{\varepsilon E_{z}}{\eta }\left(\zeta -\varphi \right)+\frac{a^{2}-r^{2}}{4\eta }\left(-\frac{dP}{dz}\right),$$

where the second term on the right-hand side is the well-known parabolic velocity profile in a cylinder, under a pressure gradient. When there is no pressure gradient-induced flow and the electrophoretic motion is measured relative to infinity where the potential value is zero, Equation (19) reduces to the following simple expression:

(20) $$u_{z}=-\frac{\varepsilon E_{z}}{\eta }\zeta .$$

Equation (20) relates the electro-osmotic fluid velocity far from the solid boundary to the zeta potential at the solid surface.

Smoluchowski used Equation (20) to great advantage in his derivation of the electrophoretic flow field. For a silica particle, the Debye layer thickness depends on the pH of the liquid but is usually smaller than or about 0.5 microns. Since $\lambda_{D}$ is small on the macroscopic scale, Smoluchowski treated it as infinitesimal. Thus, for an electrophoretic spherical particle with radius *a*, the governing electrostatic equation is $\nabla^{2}\varphi =0,$ with boundary conditions on the surface of the sphere given by $\hat{r}•\nabla \varphi {|}_{r=a}=$0, while at infinity it is given by $-\nabla \varphi =E_{\infty }$. The tangential field boundary condition at *r* = *a* follows from Equation (20), which expresses the fact that the external electric field is aligned with the velocity field as required by the electro-osmotic flow. Since the latter is parallel to the solid boundary in electroosmotic flow, Smoluchowski argued that the electric field must follow the $\hat{r}•\nabla \varphi {|}_{r=a}=0$ condition.

**Figure 5:**
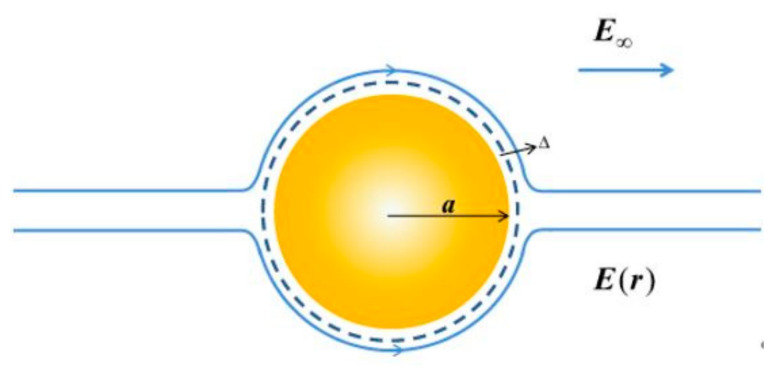
Illustration of the electric field in the outer flow region associated with the Smoluchowski argument. Here, *a* stands for the particle radius. The dashed line roughly indicates the position of the Debye layer’s outer boundary. The electric field ***E*(*r*)**→ $E_{\infty }\;$ as *r*→∞. As constructed by the Smoluchowski argument, the electric field line follows the electro-osmotic flow parallel to the solid boundary. Adapted from [19].

Since the applied uniform electric field $E_{\infty }$ represents an electric potential φ with dipolar symmetry, the solution of the Laplace equation in spherical coordinates must have the same dipolar symmetry as that of the source. When this symmetry constraint is combined with the tangential electric field boundary condition at *r* = *a*, the solution of the electric potential, expressed in terms of the electric field, is given by $E(r)=[\overset{↔}{I}+\frac{1}{2}{\left(\frac{a}{r}\right)}^{3}\left(\overset{↔}{I}-3\hat{r}\hat{r}\right)]•E_{\infty }$, where $\overset{↔}{I}$ denotes the identity matrix and $\hat{r}$ is the radial unit vector. With the generalization of Equation (20) to read as u=−(εζ/η)E, the fluid velocity field in the particle’s rest frame can be written as $u=-\frac{\varepsilon \zeta }{\eta }E_{\infty }-\frac{\varepsilon \zeta }{2\eta }{\left(\frac{a}{r}\right)}^{3}\left(\overset{↔}{I}-3\hat{r}\hat{r}\right)•E_{\infty }$. The fluid velocity component along the electric field direction is therefore given by

(21) $$u(r)•{\hat{E}}_{\infty }=-\frac{\varepsilon \zeta }{\eta }{\hat{E}}_{\infty }\cdot \left[\overset{↔}{I}+\frac{1}{2}{\left(\frac{a}{r}\right)}^{3}\left(\overset{↔}{I}-3\hat{r}\hat{r}\right)\right]•E_{\infty }=-\frac{\varepsilon \zeta }{\eta }\left[1+\frac{1}{2}{\left(\frac{a}{r}\right)}^{3}\left(1-3\cos^{2}\theta \right)\right]E_{\infty },$$

where ${\hat{E}}_{\infty }$ is the unit vector along the applied field direction. In Figure 5, we show a schematic drawing of the Smoluchowski boundary condition that yields the electrophoretic flow field.

In the laboratory frame where the fluid is at rest far from the spherical particle, we only have the second term in the square bracket of Equation (21) as the fluid flow field, which displays the functional form $u(r)•{\hat{E}}_{\infty }{|}_{lab}\sim P_{2}(\cos\theta )/r^{3}$. On the other hand, the first term in the bracket expresses the velocity of the electrophoretic particle in the laboratory frame, i.e., $v_{E}=\mu_{E}E_{\infty }$, where the electrophoretic mobility is given by $\mu_{E}=\varepsilon \zeta /\eta$ (note the sign change due to the change of the reference frame). This is the famous Smoluchowski expression, which relates the electrophoretic mobility to the zeta potential. For a negative zeta potential as that for the silica-water interface, this mobility expression means that the electrophoretic velocity is opposite to the applied field direction. This relation has largely been verified. Corrections due to the finite Debye layer thickness plus the influence of pH values have been extensively worked on over the past century, with the basic physical picture remaining intact.

### 4.3. Issues Concerning the Electrophoretic Drag Coefficient

Since $F=\gamma_{E}v_{E}$ and $v_{E}=\mu_{E}E_{\infty }$, we can combine the two expressions to yield $F=\gamma_{E}\mu_{E}E_{\infty }=Q_{E}E_{\infty }$, where $Q_{E}$ denotes the charge responsible for balancing the hydrodynamic drag under an applied field. There are two issues concerning the electrophoretic drag coefficient: the first involves the measurement of the electrophoretic drag force, and the second relates to the theoretical problem of where the interface should be located on which the electrophoretic hydrodynamic drag force and the balancing $Q_{E}$ are to be evaluated.

#### 4.3.1. The Force Measurement

Regarding the force measurement issue, there is a well-known expression [20,21] based on force balance:

(22) $$F_{ext}-\gamma_{s}(v-\mu_{E}E_{\infty })=0,$$

where $F_{ext}$ denotes the non-electric force and v denotes the particle velocity under the combined influence of electric and non-electric forces. Equation (22) suggests that the non-electric—e.g., mechanical—force needed to stall an electrophoretic motion is a Stokes-like drag force. This expression is intriguing in the sense that as long as the velocity is very close to the electrophoresis velocity, the drag force is always Stokes-like [21]. Thus, will the electrophoretic drag simply be the Stokes drag? To put this question into a sharp focus, let us re-express Equation (22) as two equations by writing $v=v_{E}+\Delta v$. Equation (22) then becomes $F_{ext}=\gamma_{s}\Delta v$ and $v_{E}=\mu_{E}E_{\infty }$. In this way, it becomes clear that the electrophoretic drag is independent from the Stokes drag, even though one has to be very careful not to contaminate the electric forcing by the non-electric components.

Experimentally, the problem of measuring the drag force was solved by using an optical tweezer to hold a single silica particle and applying an AC electric field [19]. Since the optical tweezer represents a harmonic potential trap for the held particle, the particle displacement from the center of the trap yields the force as that of a spring. Simultaneously, the velocity in a harmonic trap is always 90° out of phase with the displacement. Thus, in this manner, the force exerted by the optical trap does not interfere with the electrophoretic velocity that is actuated by the applied electric field. A more detailed analysis of this scenario is presented below, but the basic picture is that described here.

#### 4.3.2. The Relevant Surface at Which the Hydrodynamic Drag Should Be Evaluated

Provided that the hydrodynamic drag force can be measured, its value should correspond with that evaluated at a surface, and the same surface should have a net charge $Q_{E}$ to provide the balancing electrical force. Where is the location of that surface? Since the electrophoretic velocity $v_{E}$ is directly related to the zeta potential at the solid surface, should the drag force not also be evaluated at the solid surface? If that is the case, then $Q_{E}$ is simply the surface charge. The drag force evaluated in this manner can be two orders of magnitude larger than the Stokes drag, which is also consistent with the drag force estimated from the point of view of hydrodynamics. In the thin Debye layer electrophoresis, the velocity gradient in the liquid is screened beyond the $\lambda_{D}$ scale; thus, the drag force is ~$\left[(4\pi a^{2})\eta /\lambda_{D}\right]v_{E}$. As $a/\lambda_{D}$ >> 1 in most cases, it follows that $\gamma_{E}$ >> $\gamma_{S}$ on the solid surface, with the ratio of $\gamma_{E}/\gamma_{S}$ reaching as high as 100 to 200 in some cases. However, such a large drag force has never been experimentally observed, even remotely.

While the thin Debye layer approximation adopted by Smoluchowski led directly to the form of the fluid velocity field far from the solid surface, it also provided the hint to the problem solution regarding the choice of the surface on which to evaluate the hydrodynamic drag. It tells us that there must be an inner flow field which is distinct from the outer flow field. As the inner region involves net charges in the Debye layer, the mathematics of electrohydrodynamics can be highly nonlinear and difficult to solve analytically, as can be seen from the PNP equations when they are coupled to the NS equation. While there have been extensive analytical works [21,22,23,24] on the outer flow field during the past century, there was no rigorous analytic solution to the inner flow field. It is only with the appearance of commercial simulation software such as COMSOL Multiphysics that an accurate numerical solution of the problem has become possible by combining multiple modules and manually refining the finite element grid points close to the solid boundary in order to resolve the Debye layer.

The numerical solution of both the inner and outer flow fields provided a solution to the drag force problem because of the following features: (1) there is indeed a fairly sharp interface between the inner and outer flow fields; (2) the inner flow field, which extends about one to two Debye lengths from the solid surface, exhibits the electro-osmotic flow as postulated by Smoluchowski; (3) the inner flow field is carried along by the solid particle, and thus its interface with the outer flow field is logically the slip plane at which the hydrodynamic drag as well as the value of $Q_{E}$ should be evaluated. The explicit delineation of the inner flow field in electrophoretic flow thus essentially completed the work of Smoluchowski, one century after it was initiated.

The value of the drag evaluated at the inner/outer interface indeed agrees reasonably well with the measured value, on the order of two times the Stokes drag [19]. The measured value displayed a variation with the liquid pH value. Furthermore, $Q_{E}$ is much smaller than the surface charge [25,26] owing to the screening by the counter ions in the Debye layer. Simultaneously, numerical simulations also verified the relation $F_{ext}=\gamma_{s}\Delta v$ at the inner/outer interface, shown below, when a body force plus an electric field was applied (in-phase with the velocity) to the spherical colloidal particle. Therefore, the identification of the inner/outer interface represents the crucial element that closes the loop in our understanding of electrophoresis drag.

### 4.4. An Analysis of the Optical Tweezer Experiment

**Figure 6:**
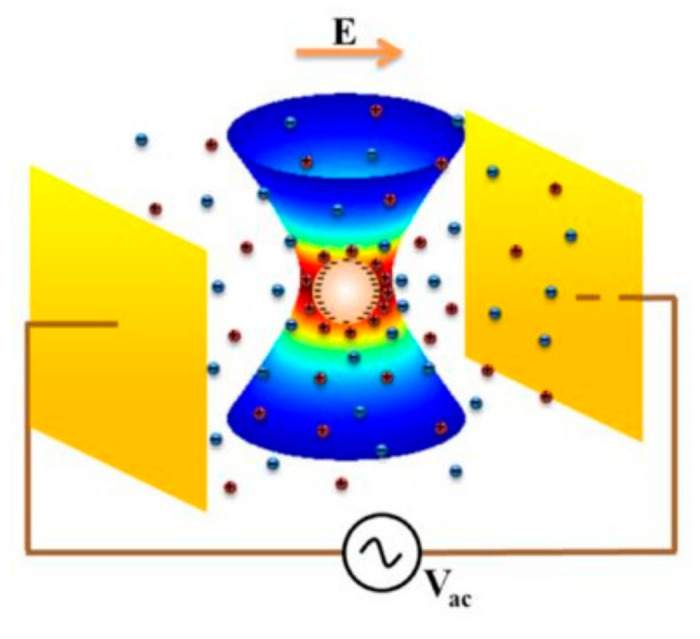
Schematic illustration of a charged colloidal particle held by an optical tweezer (with a harmonic potential) and driven by an oscillating electric field. The electric field direction is indicated at a particular instant of time. Adapted from [19].

In Figure 6, we show a schematic picture of the optical tweezer experiment setup, in which an AC electric field (20 to 100 Hz) is applied to a single spherical particle held by a calibrated optical trap. The optical trap has been calibrated to act as a harmonic potential [27], and the spring constant of the trap, $k_{trap}$, has been accurately measured [19]. The particle’s equation of motion is given by

(23a) $$m\frac{d^{2}x(t)}{dt^{2}}=-\gamma \frac{dx(t)}{dt}+E_{\infty }Q_{E}-k_{trap}x(t),$$

where *m* is the particle mass, *x* is the displacement of the particle from the center of the harmonic optical trap and γ is the drag coefficient, written purposely without a subscript since the specification of which drag coefficient applies would depend on the situation to be analyzed below.

Since $m\omega^{2}$ is five orders of magnitude smaller than $k_{trap}$, Equation (23a) can be simplified to be

(23b) $$k_{trap}x(t)=-\gamma \frac{dx(t)}{dt}+E_{\infty }Q_{E},$$

where $E_{\infty }=E_{\infty }^{\left(0\right)}\exp(-i\omega t)$. We write the displacement of the electrophoretic particle as $x(t)=D(\omega )e^{-i[\omega t-\delta (\omega )]}$, with the displacement amplitude denoted by D(ω) and the phase δ(ω) defined relative to the applied AC electric field. The two parameters D(ω) and δ(ω) represent the experimentally measured quantities. By substituting the particle displacement expression for *x*(*t*) and the associated displacement velocity dx(t)/dt back into Equation (23b) and taking the real part, we obtain

(24) $$k_{trap}D(\omega )\cos[\omega t-\delta (\omega )]=\gamma \omega D(\omega )\sin[\omega t-\delta (\omega )]+E_{\infty }^{\left(0\right)}Q_{E}\cos(\omega t).$$

Let us define a $t_{0}$ such that $\omega t_{0}=\delta (\omega )+\frac{\pi }{2}$, and let $\omega t=\omega (t_{0}+t^{\prime })=\omega t_{0}+\omega t^{\prime }$. Equation (24) can be re-written in the following form:

(25) $$k_{trap}D(\omega )\sin(\omega t^{\prime })=-\gamma \omega D(\omega )\cos(\omega t^{\prime })+E_{\infty }^{\left(0\right)}Q_{E}\sin[\omega t^{\prime }+\delta (\omega )].$$

At $t^{\prime }$ = 0, the left-hand side vanishes—i.e., the optical trap force is zero—and we only have the right-hand side, from which we obtain

(26) $$\gamma_{E}\omega D(\omega )=E_{\infty }^{\left(0\right)}Q_{E}\sin[\delta (\omega )].$$

Here, we label the drag coefficient as $\gamma_{E}$ because there is only an electric field force present in Equation (26). Now, let us expand $\sin[\omega t^{\prime }+\delta (\omega )]=\sin(\omega t^{\prime })\cos[\delta (\omega )]+\cos(\omega t^{\prime })\sin[\delta (\omega )]$ in Equation (25). Then, Equation (25) can be re-organized in a physically clear manner as

(27) $$[k_{trap}D(\omega )-E_{\infty }^{\left(0\right)}Q_{eff}\cos\delta (\omega )]\sin(\omega t^{\prime })=[-\gamma_{E}\omega D(\omega )+E_{\infty }^{\left(0\right)}Q_{eff}\sin\delta (\omega )]\cos(\omega t^{\prime }).$$

In Equation (27), the right- and left-hand sides represent the in- and out-of-phase components, respectively. There is no approximation made in deriving Equation (27); thus, it has to be valid for all values of $t^{\prime }$. The only way that Equation (27) can be true is that both sides must separately be zero, since the time variations on the two sides are orthogonal to each other.

Physically, Equation (27) tells us that the electrical force can be viewed as comprising two components. One component, $E_{\infty }^{\left(0\right)}Q_{E}\sin\delta (\omega )\cos(\omega t^{\prime })$, gives rise to the time-varying electrophoretic velocity $\omega D(\omega )\cos(\omega t^{\prime })$ with a constant drag coefficient $\gamma_{E}$. The other component, $E_{\infty }^{\left(0\right)}Q_{E}\cos\delta (\omega )\sin(\omega t^{\prime })$, counterbalances the optical trap force $k_{trap}D(\omega )\sin(\omega t^{\prime })$. This optical trap response force is out of phase with the electrophoretic velocity, in contrast to the external force in Equation (22), which is in-phase with the electrophoretic velocity.

Equation (27) essentially expresses the fact that 0 = 0, from which we obtain two independent equations:

(28a) $$k_{trap}D(\omega )=E_{\infty }^{\left(0\right)}Q_{E}\cos\delta (\omega ),$$

(28b) $$\gamma_{E}\omega D(\omega )=E_{\infty }^{\left(0\right)}Q_{E}\sin\delta (\omega ).$$

By dividing (28b) by (28a), we obtain the consistency condition imposed by the force balance of the in- and out-of-phase components of the AC experiment:

(29a) $$\gamma_{E}=\frac{k_{trap}\tan\delta (\omega )}{\omega }.$$

Equation (28a) can be re-written for $Q_{E}$ in terms of the physically measured quantities as

(29b) $$Q_{eff}=\frac{k_{trap}D(\omega )}{E_{\infty }^{\left(0\right)}\cos\delta (\omega )}.$$

From Equations (29a,b), the mobility is obtained in terms of the experimental parameters as

(29c) $$\mu_{E}=Q_{E}/\gamma_{E}=\frac{\omega [D(\omega )/E_{\infty }]}{\sin[\delta (\omega )]}.$$

We refer the readers to the work presented in [19] for experimental details, but a clean resolution of the hydrodynamic drag force measurement issue is apparent from the above description.

### 4.5. Features of the Inner Flow Field

**Figure 7:**
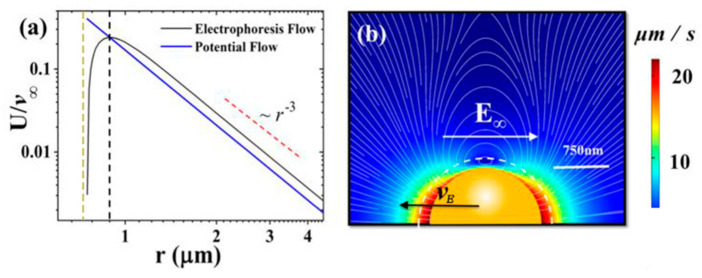
Projected velocity and streamlines in the laboratory frame for a 0.75 µm radius charge particle under a stationary condition (force balance with no acceleration). The white bars in the figures denote the length scale of 750 nm. (**a**) *P*_2_ projection of the electrophoretic velocity component along the electric field direction, denoted by U normalized by the electrophoresis velocity $v_{\infty }=\mu_{E}E$, showing very good far-field 1*/r*^3^ behavior, as indicated by the red dashed line. The yellow dashed line shows the position of the particle surface. The blue line shows the *P*_2_ projection of the Smoluchowski potential flow velocity field. It is clear that the projected 1*/r*^3^ behavior stops a small distance away from the solid surface. The peak position of the projected result is delineated by the black dashed line, corresponding to the white dashed curve in panel (**b**). (**b**) Streamlines for the negatively charged polystyrene sphere with *a* = 0.75 µm (with $\lambda_{D}$ = 96.1 nm, *ka* = 7.8, *S*= -7000 *e*/µm^2^) plotted in the lab frame. Here, the colors indicate the magnitude of the velocity, while the interface between the inner and outer flow fields (the reference surface) is denoted by the white dashed curve. Adapted from [19].

We first show how the inner flow field and its interface with the outer flow field can be identified. By projecting the $P_{2}(\cos\theta )$ angular profile on the numerically simulated solution, one obtains the $1/r^{3}$ behavior along the radial direction, as shown in Figure 7a, which agrees very well with the Smoluchowski solution’s radial behavior in the outer flow field, also shown here as the blue line. However, this agreement stops abruptly at a distance, ~two Debye lengths from the solid surface, at which point the projected value U drops precipitously, indicating the fact that the flow field no longer agrees with the outer flow field solution of Smoluchowski (blue line), which goes all the way to the solid surface. The maximum value of U is therefore identified as the interface between the inner flow field and the outer flow field.

In Figure 7b, we show the details of the inner flow field. The most prominent feature is the belt of vortices surrounding the equatorial plane of the spherical particle, if the poles are identified to be along the direction of ${\hat{E}}_{\infty }$. The vortices are clearly driven by the electro-osmotic flow induced by the net charges in the Debye layer, with the drag of the solid surface (via the no-slip hydrodynamic boundary condition) along the opposite direction being responsible for the counter flow in the vortex belt. It is clear from this picture that the slip surface between the solid particle and the fluid must lie in the neighborhood of the inner/outer interface, with the inner flow field being carried along by the solid particle. A quantitative confirmation of this point is given below.

It should be noted that the belt of vortices only exists in the laboratory frame, where there is velocity reversal in the inner flow field. In the frame in which the particle is at rest, there is no velocity reversal; thus, no vortices belt can be seen. The other requirement for the appearance of vortices is of course the existence of a vorticity source in the NS equation. This is addressed in the following section.

### 4.6. The Electro-Hydrodynamic Vorticity Source in the Inner Flow Field

In hydrodynamics, it is well known that, besides the requirement of velocity reversal, there is also a need for a vorticity source in the formation of vortices. The traditional source is the nonlinear term in the NS equation, $\rho_{l}(u•\nabla )u$, the magnitude of which is characterized by the dimensionless Reynold’s number Re $=\rho_{l}ud/\eta$. In the present case, since the relevant Reynold’s number is on the order of $10^{-6}$, this vorticity source is negligible. Thus, there must be another vorticity source that gives rise to the observed belt of vortices. That source should be electro-hydrodynamic in nature since the inner flow field has a high concentration of net charges.

Vorticity is defined as ω=∇×u. For viscous incompressible flow with electrical body force, the Navier–Stokes equation is given by

(30a) $$\frac{\partial u}{\partial t}+u•\nabla u=-\frac{1}{\rho_{l}}\nabla P+\frac{\eta }{\rho_{l}}\nabla^{2}u+\frac{1}{\rho_{l}}f,$$

where ***f*** =$-e(n_{+}-n_{-})\nabla \varphi$. By taking the curl of both sides of the NS equation and using the Laplacian of the electrical potential to eliminate the net charge (via the Poisson equation), the steady-state dimensionless vorticity equation with electrical body force can be written as

(30b) ∇2ω¯+Reω¯•∇u¯+(u/vE)ζE∞d∇∇2φ¯×∇φ¯=0,

where $\bar{u}=\frac{u}{v}$, $\bar{l}=\frac{l}{d}$, $\bar{t}=\frac{t}{d/v}$, $\bar{\omega }=\frac{\omega d}{v}$ and $\bar{\varphi }=\frac{\varphi }{\zeta }$ are the non-dimensionalized variables. If we ignore the second term on the left hand side of Equation (30b) due to its negligible magnitude, the third term is seen to serve as a source for the vorticity, characterized by a new dimensionless number $\Xi =(u/v_{E})(\zeta /E_{\infty }d)$. If we approximate $u/v_{E}\approx 1$ in the vicinity of the solid boundary, then $\Xi ≅\zeta /E_{\infty }d$. For ζ~25mV, *d* ~ 1 μm, and $E_{\infty }$ *=* 500 V/m, we have Ξ=50, which is large enough to serve as a substantial source of vorticity. It should be noted that Ξ vanishes quickly outside the Debye layer, with *u* approaching the far-field values.

### 4.7. Stokes Drag vs. Electrophoretic Drag on the Interface of the Inner/Outer Flow Fields

**Figure 8:**
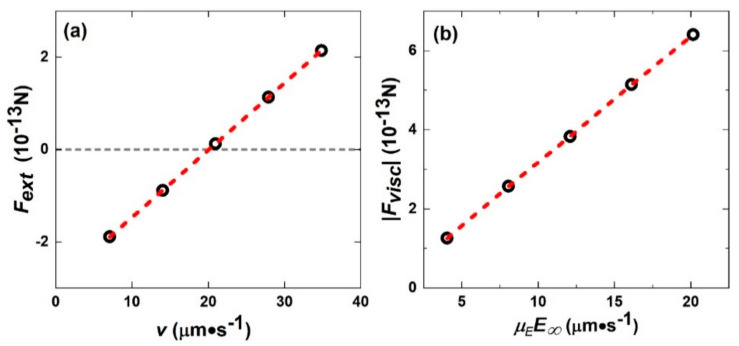
The difference between $\gamma_{S}$ and $\gamma_{E}$. (**a**) The external force *F*_ext_ at the inner/outer interfacial region shows a linear dependence with the relative velocity *v* between the sphere and simulation boundary (infinity). The open circles indicate five different values of *v*. The red dashed line denotes the best fitting with a slope of 1.45 *×* 10*^−^*^8^ kg*·*s*^−^*^1^, which is very close to the Stokes drag coefficient 1.41 *×* 10*^−^*^8^ kg*·*s*^−^*^1^ (=6*πηa,* 3% lower than the slope). The external force becomes zero when *v* is exactly at the electrophoretic velocity. The external electric field strength was maintained at $E_{\infty }$ = 500 V/m. (**b**) The viscous force *F*_visc_ at the inner/outer interfacial region with a relative velocity $v=\mu_{E}E_{\infty }$ between the sphere and the liquid at the simulation boundary (infinity). The external electric field strength was varied from 100 V/m to 500 V/m in 100 V/m steps. The red dashed line indicates fitting with a slope of 2.9 *×* 10*^−^*^8^ kg*·*s*^−^*^1^ (=2.1 *×* 6*πηa*). All simulations were performed with a surface charge density of *S* = *−*7 *×* 10^3^ *e*/µm^2^, radius of *a* = 0.75 µm and ionic strength of 0.01 mM ($\lambda_{D}$ = 100 nm). Adapted from [19].

In [19], simulations were carried out to accurately evaluate the drag coefficients on the inner/outer interface, both to check the veracity of Equation (22), $F_{ext}-\gamma_{s}(v-\mu_{E}E_{\infty })=0$, as well as to obtain the electrophoretic drag when the in-phase $F_{ext}$ is absent. All simulations were performed with a spherical particle with a radius of *a* = 0.75 μm, a surface charge density of $S=-7\times 10^{3}e{/\mu m}^{2}$ and an ionic strength of 0.01 mM (*λ*_D_ = 100 nm). By keeping the solid particle stationary and varying the boundary value of the far-field fluid velocity ***u***, we evaluate the magnitude of $F_{ext}$ as a function of ***u***, with the externally applied electric field maintained at $\left|E_{\infty }\right|$ = 500 V/m. In Figure 8a, we show that the external force $F_{ext}$ displays a linear dependence on the relative velocity between the solid particle and the bulk fluid, with a slope given by 1.03$\gamma_{S}$. Thus, Equation (22) is verified, i.e., indeed $\gamma_{S}=F_{ext}/\Delta v$ at the interface between the inner and outer flow fields.

In Figure 8b, we focus on the case in which $F_{ext}=0$. By evaluating the viscous drag force, $F_{visc}$, at the inner/outer interface and varying the external electric field strength from 100 V/m to 500 V/m in 100 V/m steps, we plot the resulting $F_{visc}$ as a function of the electrophoretic velocity $\mu_{E}E_{\infty }$. The slope gives a value that is 2.1 times the Stokes drag coefficient, which is somewhat higher than the experimentally measured value of $\gamma_{E}/\gamma_{S}=1.7\pm 0.3$ but definitely out of the range of the Stokes drag.

## 5. Concluding Remarks

In this article, we have reviewed two classical issues in electrokinetics, both fundamental in nature, with new viewpoints and physical pictures that have only recently become available. It is our hope that the enhanced understanding of the Poisson–Boltzmann equation and the newly available physical picture of electrophoresis can further propel investigations on the challenging topic of charged interfaces.
